# Effects of the Excessive Use of Sugar on the System

**Published:** 1864-12

**Authors:** 


Effects of the Excessive Use of Sugar on the System.—Dr. Champouillon communicated the result of his observations on the effects of the excesssive use of sugar on the system. So far back as the year 1846, the author undertook a series of experiments on himself, in order to supply the Minister of War with information as to the possibility of replacing salt by sugar in the preparation of the preserved meat destined for the use of the army during a campaign. In accordance with his instructions, M. Champouillon strictly confined himself to the diet which may be accidentally enforced on the garrison of a besieged city by the hardships of war, and for several days in succession lived on the following rations: sixteen ounces of beef preserved in sugar, and four ounces of biscuit; water was his only beverage. Various phenomena supervened in the following: thirst, sinking at the stomach, distaste for food, nausea, acid regurgitation, epigastric pain, diarrhœa, prostration, and syncope.

"I carefully watched these symptoms," says M. Champouillon, "and the loss of appetite and nausea indubitably proceeded from the absence of variety in my diet; whereas the thirst, heartburn, epigastric pain and diarrhœa were as clearly referable to the difficulty of digesting cane sugar. In proportion to the impression produced by this substance on the organs of taste, it clogs the palate and destroys natural appetite. This excessive indulgence in syrups, sweetmeats, pastes, and highly-sweetened diet-drink, brings on distaste for food, and annihilates the digestive powers, especially in cases of pulmonary consumption. After expatiating on the transformation of cane-sugar into glucose, in consequence of its contact with the acids contained in the gastric juice, and on the injury caused by the increased activity imparted to the functions of the stomach by frequent repetition of the process, M. Champouillon showed that in addition to the inflammatory congestion thus occasioned glucose powerfully contributes to the establishment of a plethoric condition of the system, and that the prevalent opinion that the excessive use of sugar tends to cause pulmonary irritation and a disposition to atrophy, is but too well justified by facts. In support of this view, the author adduced two interesting cases; one of apoplexy, the other of hæmoptysis, in which the agency of this cause was distinctly evident.

"I have often remarked," said he, "in thirty years' experience of tubular disease, that the cough, hectic fever, and night-sweats are increased by the fondness of the patients for sweet substances. I conceive this to be the natural consequence of the combustion of the glucose in the system, a phenomenon which necessarily implies the production of water, carbonic acid, and heat. It is a well-known fact that three and a half ounces of sugar consumed in the human body evolve an amount of heat equivalent to what might be produced by the combustion of thirty-two grains of charcoal. MM. Favrot and Silberman have shown that fifteen grains of charcoal are sufficient to impart one degree (cent.) of heat, to eight kilogrammes, or sixteen pounds of water. If the capacity of the human body for caloric is the same as that of water, three ounces and a half of sugar will, in a subject weighing seventy-five kilogrammes (12½ st.) raise during their combustion the temperature of the body four degrees and a half (centigr).

The practical conclusion of this paper is that it is desirable to reduce within as narrow limits as possible the consumption of sugar, especially in cases of tuberculosis, and to replace that substance by honey, or a decoction of liquorice.—[*Dublin Med. Press*, Feb. 24, 1864, from *Journal de Med. et Chirurg.*

